# Baclofen for stroke patients with persistent hiccups: a randomized, double-blind, placebo-controlled trial

**DOI:** 10.1186/1745-6215-15-295

**Published:** 2014-07-22

**Authors:** Cuie Zhang, Ruifen Zhang, Shuangyan Zhang, Meiling Xu, Shuyan Zhang

**Affiliations:** 1 Department of Neurology, Fourth Affiliated Hospital of Harbin Medical University, Harbin 150001, China; 2 Department of Neurology, Fourth Affiliated Hospital of Harbin Medical University, No.37 Yiyuan Street, Nangang District, Harbin Heilongjiang Province 150001, China

**Keywords:** Baclofen, Stroke, Hiccups: Randomized controlled trial

## Abstract

**Background:**

The results of preclinical studies suggest that baclofen may be useful in the treatment of stroke patients with persistent hiccups. This study was aimed to assess the possible efficacy of baclofen for the treatment of persistent hiccups after stroke.

**Methods:**

In total, 30 stroke patients with persistent hiccups were randomly assigned to receive baclofen (*n* = 15) or a placebo (*n* = 15) in a double-blind, parallel-group trial. Participants in the baclofen group received 10 mg baclofen 3 times daily for 5 days. Participants assigned to the placebo group received 10 mg placebo 3 times daily for 5 days. The primary outcome measure was cessation of hiccups. Secondary outcome measures included efficacy in the two groups and adverse events.

**Results:**

All 30 patients completed the study. The number of patients in whom the hiccups completely stopped was higher in the baclofen group than in the placebo group (relative risk, 7.00; 95% confidence interval, 1.91–25.62; *P* = 0.003). Furthermore, efficacy was higher in the baclofen group than in the placebo group (*P* < 0.01). No serious adverse events were documented in either group. One case each of mild transient drowsiness and dizziness was present in the baclofen group.

**Conclusions:**

Baclofen was more effective than a placebo for the treatment of persistent hiccups in stroke patients.

**Trial registration:**

Chinese Clinical Trials Register: ChiCTR-TRC-13004554

## Background

Hiccups are caused by involuntary multiple spastic contractions of the diaphragm and intercostal muscles. This action is rapidly accompanied with uncontrollable inhalation and a sudden closure of the respiratory tract by the epiglottis, resulting in the classic “hic” sound [1,2]. Episodes of hiccups often start with sudden inspiration and end with abrupt closure of the glottis. Although hiccups are thought to develop through the hiccup reflex arc, the pathophysiology of hiccupping is still poorly understood. Hiccups are classified under three categories depending on their duration: acute, persistent, and intractable hiccups. Acute hiccups are defined as a hiccupping episode that lasts for minutes to hours; persistent hiccups last for more than 48 hours; and intractable hiccups last for more than 1 month. Hiccups are not only confined to adults but are also observed among infants [3,4] and children [5].

Stroke patients experience a variety of symptoms and complications that can significantly impair their quality of life. Some of the most commonly encountered complications in clinical practice include pressure ulcers [6-8], depression [9], motor disability [10], insomnia [11], and hiccups [12].

A wide range of pharmacologic interventions has been used to treat persistent and intractable hiccups, such as baclofen [13-15], gabapentin [16], chlorpromazine [17], haloperidol [18], and metoclopramide [19]. Several clinical studies have reported that baclofen may help treat persistent hiccups occurring after a stroke [13-15]. Although a Cochrane systematic review found no sufficient evidence for the treatment of persistent or intractable hiccups with either pharmacologic or nonpharmacologic interventions, all four studies included in the review had focused on acupuncture, and not baclofen, or even other pharmacologic interventions [20]. To the best of our knowledge, only a few randomized controlled trials with small sample sizes have evaluated the efficacy of baclofen for persistent hiccups after stroke.

We conducted a randomized, double-blind, placebo-controlled, 5-day clinical trial to evaluate the possible benefit of baclofen in treating persistent hiccups after stroke. We primarily hypothesized that baclofen is superior to placebo for stroke patients with persistent hiccups.

## Methods

### Study design

This study was a two-parallel arm, blinded, randomized controlled trial. The trial was conducted in accordance with the Declaration of Helsinki and the Guidelines for Good Clinical Practice at the Evidence-Based Medicine Center of the Fourth Affiliated Hospital of Harbin Medical University, a clinical research center in China. Patients were recruited between August 2012 and November 2013. The study was approved by the Medical Ethics Committee of the Fourth Affiliated Hospital of Harbin Medical University. Eligible participants were randomly allocated to the baclofen group or the placebo group at a 1:1 allocation ratio and received treatment for 5 days, with 15 days of follow-up. This study complied with CONSORT guidelines.

### Inclusion and exclusion criteria

We included participants aged 18 to 65 years with persistent hiccups (more than 48 hours but less than 1 month) after stroke. In addition, participants were required not to have taken baclofen within 15 days before study entry and were required to sign an informed-consent form.

The exclusion criteria included persistent hiccups mainly associated with cancer [21], multiple sclerosis [22], meningitis [23], brain abscess [24], traumatic brain injury [1], encephalitis [25], spinal cord lesions [26], kidney failure [27], pneumonia [28], laryngitis [29], and cardiorespiratory arrest [30]; rejection of baclofen therapy; and failure to complete clinical treatment.

### Randomization

Randomization was performed by using a computer, and group-allocation instructions were concealed in opaque sealed envelopes. For assignment to the baclofen or placebo group, participants were required to select one of the sequentially numbered envelopes.

### Participants and recruitment

We planned to conduct our research in the Fourth Affiliated Hospital of Harbin Medical University. In preparing for this research, we found that this center had offered baclofen or placebo treatment to 30 people between August 2012 and November 2013. This enabled a fair test of the feasibility criteria, and if recruitment was good, potentially permitted a reliable calculation of the effect size of the intervention for computing the subsequent sample size. Those individuals who elected to undergo baclofen treatment were informed about the research and given an information sheet. From those agreed to participate, consent was given at the next appointment. After the clinical assessment, participants were randomized to receive baclofen or a placebo, both of which were given by therapists, who were trained in their administration.

### Intervention

Participants recruited to the baclofen group received 10 mg 3 times daily for 5 days. Participants assigned to the placebo group received 10 mg of a placebo 3 times daily for 5 days. The participants were then asked to identify any obstacles to the goal attainment and to consider the possibility that the therapy could help them overcome these obstacles. This was intended to motivate the participants to engage in the therapy.

The placebo tablets used in this study were composed of hydroxypropyl cellulose, pregelatinized starch, dextrin, starch, silicon dioxide, magnesium stearate, sodium carboxymethyl starch, sucrose, talcum powder, gelatin, silicone oil, and Chinese wax. The tablets were produced by Changzhou Siyao Pharmaceuticals Co. Ltd. and had the same dose, taste, and color as baclofen.

### Assessments

Patients outcomes were evaluated by using the Chinese Medicine Medical Association standard criteria [12] and classified as follows: cure, cessation of persistent hiccups within the intervention period, with no relapse in one week; improvement, reduction in the frequency and severity of hiccups and other symptoms (such as abdominal discomfort); and no effect, no amelioration of hiccups.

### Data collection and analysis

In addition to the clinical outcomes, the efficacy and side effects in the two groups were recorded and analyzed. We collected data on the number of eligible participants with persistent hiccups, willingness of the participants to be randomized, and compliance with the intervention, and aimed to estimate the effect size for a fully powered trial.

### Sample size and analysis

This feasibility study estimated that a sample of 30 participants would be sufficient to provide data to answer our study questions [31]. Clinical outcome data were analyzed by using an “intention-to- treat” approach, and the initial analysis examined the demographic and baseline characteristics of patients randomized to the trial. Between-group differences in categoric data were assessed by using the Fisher Exact test or Mann–Whitney *U* test; the *t* test was used for continuous data. Relative risks and 95% confidence intervals were also reported. Levels of significance were reported at *P* < 0.05. Descriptive statistics were used to assess the feasibility questions. Analysis was performed by a statistician who was blinded to the study group.

## Results

**Figure 1:**
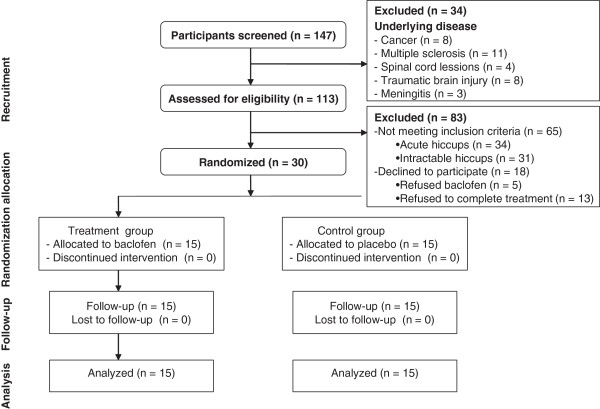
Flow chart of participants through the trial.

In this study, 147 participants were initially screened. Of these 147 people, 34 subjects were excluded because of the following: cancer (eight patients), multiple sclerosis (11 patients), spinal cord lesions (four patients), traumatic brain injury (eight patients), and meningitis (three patients). The remaining 113 patients were entered into the study. Of these 113 patients, 65 did not meet study criteria (34 patients had acute hiccups, and 31 patients had intractable hiccups), and 18 declined to participate (five patients refused baclofen; 13 patients failed to complete treatment). Therefore, 30 individuals were randomized into the study. All 30 participants completed the study and were included in the final analysis (Figure 1).

The characteristics of the study sample are presented in Table 1. The two groups did not differ significantly in the majority of sociodemographic and clinical variables investigated at the baseline. At the baseline, the mean age was 60.38 ± 19.61 years in the baclofen group and 58.64 ± 18.57 years in the placebo group. The duration of hiccups was 59.24 ± 9.65 hours and 61.32 ± 10.34 hours in the baclofen group and placebo group, respectively. All patients had a history of stroke. In the baclofen group, 10 and five patients had had ischemic and hemorrhagic strokes, respectively, whereas in the placebo group, nine and six participants had had ischemic and hemorrhagic strokes, respectively. The ischemic and hemorrhagic strokes had occurred 3.71 ± 1.28 months and 3.83 ± 1.35 months ago, respectively, in the baclofen group, and 3.65 ± 1.32 months and 3.87 ± 1.39 months ago, respectively, in the placebo group. The Fugl–Meyer Assessment (FMA), modified Barthel Index (MBI), and Neurological Deficit Scale (NDS) scores were 49.56 ± 21.67, 50.97 ± 24.15, and 15.12 ± 5.89, respectively, in the baclofen group, and 51.21 ± 23.11, 52.24 ± 23.53, and 13.96 ± 5.77, respectively, in the placebo group.

**Table 1:** Baseline characteristics of participants at trial entry

	**Variable**	**Group**		***P *** **value**
		**Baclofen (*n*** **= 15)**	**Placebo (*n*** **= 15)**	
Age	Mean (SD)	60.38 (19.61)	58.64 (18.57)	0.78
Gender				
	Male	8 (53.33%)	9 (60.00%)	0.71
	Female	7 (47.67%)	6 (40.00%)	0.71
Married		15 (100%)	14 (93.33%)	0.47
Ethnicity				
	Han Chinese	14 (93.33%)	13 (86.66%)	0.55
	Other	0 (0.0%)	1 (6.67%)	0.49
	Not recorded	1 (6.67%)	1 (6.67%)	1.00
Employment				
	Employed	2 (13.33%)	1 (6.67%)	0.55
	Unemployed	1 (6.67%)	1 (6.67%)	1.00
	Retired	12 (80.00%)	13 (86.66%)	0.63
Education				
	Completed high school	12 (80.00%)	13 (86.66%)	0.63
	Completed tertiary education	3 (20.00%)	2 (13.34%)	0.63
Duration of hiccups (hours)	Mean (SD)	59.24 (9.65)	61.32 (10.34)	0.51
Stroke				
	Ischemic	10 (66.67%)	9 (60.00%)	0.71
	Hemorrhagic	5 (33.33%)	6 (40.00%)	0.71
Duration of stroke (months)	Mean (SD)			
	Ischemia	3.71 (1.28)	3.65 (1.32)	0.90
	Hemorrhage	3.83 (1.35)	3.87 (1.39)	0.94
Severity of stroke	Mean (SD)			
	FMA	49.56 (21.67)	51.21 (23.11)	0.84
	MBI	50.97 (24.15)	52.24 (23.53)	0.88
	NDS	15.12 (5.89)	13.96 (5.77)	0.59

FMA, Fugl-Meyer Assessment; MBI, Modified Barthel Index; NDS, Neurological Defect Scale.

An analysis of the clinical outcomes is presented in Table 2. Fourteen participants in the baclofen group were cured, compared with two in the placebo group (RR, 7.00; 95% CI, 1.91–25.62; *P* = 0.003). One participant in the baclofen group showed improvement in persistent hiccups, compared with five patients in the placebo group (RR, 0.20; 95% CI, 0.03–1.51; *P* = 0.12). None of the patients in the baclofen group had no amelioration of the hiccups, compared with eight patients in the placebo group (RR, 0.06; 95% CI, 0.00–0.94; *P* = 0.04). In addition, a significant difference in efficacy was found between the two groups (*P* < 0.01; Table 3).

**Table 2:** Comparison of outcome measures between the two groups

**Outcomes**	**Baclofen (*n*** **= 15)**	**Placebo (*n*** **= 15)**	**Relative risk (95% CI)**	***P *** **value**
Hiccup cessation	14	2	7.00 (1.91–25.62)	0.003
Improvement	1	5	0.20 (0.03–1.51)	0.120
No effect	0	8	0.06 (0.00–0.94)	0.040

**Table 3:** Comparison of efficacy between the two groups

**Group**	**Total patients**	**Hiccup cessation**	**Improvement**	**No effect**
Baclofen	15	14	1	0
Placebo	15	2	5	8

Difference in efficacy between the two groups, *P <* 0.01.

No serious adverse events related to treatment were documented, which might be due to the short treatment duration. However, two patients in the baclofen group reported mild side effects. One patient reported mild transient drowsiness, and the other reported mild dizziness.

## Discussion

Our study provides data indicating that further evaluation of baclofen is warranted in stroke patients with persistent hiccups. Analysis of the clinical outcomes identified trends in the cessation of persistent hiccups within the intervention period; however, the numbers were too small, with wide confidence intervals. The study demonstrated the acceptability of baclofen by the subjects, randomization, and participation in the trial. Compliance and follow-up were also acceptable. Recruitment was slower than planned, and barriers to recruitment included the low prevalence of persistent hiccups among stroke patients and recruitment at a single hospital site.

Our results suggest that 10 mg baclofen 3 times daily is an acceptable dose, and concur with the findings of Mirijello [13]. Both studies found that persistent hiccups can be successfully treated with baclofen without any significant side effects. Our study highlighted the need for further research on the topic. Findings from the physician interviews may not represent the views of all clinicians at the host institution or be considered representative of physicians elsewhere; however, our findings indicate a supportive research culture for this topic of study.

The side effects were generally well tolerated in this study. No serious adverse events were recorded, and only two patients reported mild side effects. One patient reported mild transient drowsiness, and the other reported mild dizziness. This low incidence of adverse effects might be attributable to the short duration of the study.

The study has several strengths. First, the trial was randomized, thereby reducing selection bias. Second, although no consensus was found on the optimal dose of baclofen for persistent hiccups, our findings suggest that the dose used in this study was in the therapeutic range. However, the main limitation of the present study is its size, and our findings should therefore be interpreted with caution.

The findings of this study will influence future decisions concerning resources and planning for trials. We consider that the randomization was acceptable, and we will explore whether the window for eligibility can be extended. An appropriate sample size has been determined to allow for attrition. Therefore, further studies with larger numbers of patients receiving baclofen are required to verify the results of this study.

## Conclusions

The results of this study provide evidence to support the hypothesis that baclofen may be of some use in treating persistent hiccups. However, larger studies are warranted.

## Abbreviations

ChiCTR: Chinese Clinical Trials Register; CI: confidence interval; FMA: Fugl–Meyer Assessment; MBI: Modified Barthel Index; NDS: Neurological Deficit Scale; RR: Relative risk.

## Competing interests

The authors declare that they have no competing interests.

## Authors’ contributions

SZ (Shuangyan Zhang) conceived of the study, participated in the coordination and design of the study, performed. CZ performed the statistical analysis and wrote the paper. RZ carried out the clinical assessment and participated in most parts of the study (main performer). MX, and SZ(Shuyan Zhang) participated in the coordination of the study. All authors read and approved the final manuscript.
